# Eight Letters with Replies

**Published:** 1874-07

**Authors:** 


### Our Correspondence.

### Letters Exposing Humbugs and Swindlers,

**With other Communications of Interest to the People.**

ONAWA, Iowa, March 16, 1874.

THAD. S. UP DE GRAFF, M. D.

*Dear Sir:*—As I am asked about one Dr. J. Ball & Co., 19 Liberty street, New York city, who advertises a patent Ivory and Lignum Vitæ Eye Cup for restoring lost sight, weak eyes, &c., I have concluded to write to you for your opinion of it, in the next BISTOURY. What I most admire in this BISTOURY of yours, is the exposition of quacks and humbugs. I suppose you must know something of this Dr. Ball, as he sends lots of his papers out here. Truly yours, J. B., M. D.

For our opinion, in full, of the Eye Cup swindle, we refer our readers to the BISTOURY of April, 1872.

---

KIOWA AND COMANCHE INDIAN AGENCY,

Near FT. SILL, Ind. Ter.,

5TH, 5, 1874.

T. S. UP DE GRAFF, M. D.

*Dear Sir:*—Enclosed find fifty cents for BISTOURY. I am so well pleased with your journal, I feel that I cannot do without it. I found one recipe in the October number, which I value at more than ten times the cost of the journal. Truly yours,

O. G. GIVEN, M. D.

Care Agent HAWORTH.

Glad to note your appreciation of the BISTOURY, Doctor. We give you many valuable formulæ, in the present number, which would have cost much money and considerable time to have collected by any other means. It affords us much satisfaction to know that our medical brethren place great value upon our medical items and news.

---

TROY, Pa., April 9, 1874.

DR. UP DE GRAFF:

*Dear Sir:*—Can you give me any insight into the virtues of the scrofula cure, zozo, hair tonic, Jamaica rum, cherry pectoral, &c., sold by J. Ball & Co., of New York? There is a woman here selling them, and claiming to cure all the diseases flesh is heir to. She sells plenty of this trumpery, but cures no one. Are they humbugs, swindlers, or what in the name of common sense are they ? If you can give any account of them, you will confer a great favor on the community in general. Yours, &c., C. J. C.

Now, there's a poser ! Give any *insight* to such a batch of quack nostrums ! Why, our correspondent should carefully keep in sight the fact, that all patent medicines are useless as curative agents ; that traveling doctors are usually imposters ; and that migratory medicine peddlers are simply vagabonds, too lazy to work or steal, but just shrewd enough to bamboozle those innocent mortals who would likely invest in J. Ball & Co.'s sham decoctions. Are they humbugs ?—Shades of the immortal Sanco Panza—Y-E-S!

---

BINGHAMTON, June 15, 1854.

DEAR BISTOURY :

What is good for a felon ? B.

Take him to the penitentiary.

---

ROME, N. Y., June 1, 1874.

DR. UP DE GRAFF :

They say you are good for everything—please tell me what to do for chapped lips, and oblige, A YOUNG LADY.

No, you don't ! You want us to say something smart, don't you ? "They say"—*do* they, though ?—Why, confound it, we're married, and what is more, got four big boys. *Ergo*—can't make the reply you expected, though the homœpathic law would apply beautifully in your case, (applying Chap to chap), provided your face is as handsome as your letter. Sorry, but we positively dare not prescribe in this instance.

---

DR. UP DE GRAFF :

*Sir :*—My age is 45, and I am very much annoyed by what you call *muscæ volitantes*, especially in the right eye, which prevents my reading figures quickly. The obstruction appears as two minute black spots ; also like small clouds of mucilage ; and then like small translucent worms or flexible hairs. If my bowels are not free, they seem to be denser and the pupil more contracted. Three years ago I was examined carefully by an oculist, who prescribed corrosive sublimate, but I perceived no good effect therefrom.

I shall be obliged for a few words of advice.

Yours, J. M. T.

Read the article upon *Musicæ Volitantes* in present number.

GLASGOW, MO., May 9th, 1874.

DR. UP DE GRAFF :

My little daughter and myself are still getting additional names for your valuable BISTOURY. Hope next time to swell the number considerably. They all want to commence with the April No. Your article on the "Prevailing Excitement," even our Good Templar folks read and approved of. It has been read in a respectable lodge of about one hundred members here.

Yours truly, in haste,

B. E. POWELL, M. D.

Glad to hear your "Good Templars" are sound on the Crusading question.

---

PALMYRA, MO., May 20, 1874,

ED. BISTOURY :

Will you be good enough to give me some cures for warts in the next number of the BISTOURY ? L.

Certainly. Let us see, perhaps we can call to mind those to which we have heard great virtues ascribed: Yes, we've got it !—Steal —mind you, *steal* a piece of fat pork, taken from the left, fore ham of a black pig. [Perhaps they don't cut their hams from the shoulder now, they used to when *we* lived West.] Well, rub your wart or warts with the aforementioned pork, then bury it under the eaves of the house, at midnight. On the ninth day, not a wart will you have. (nor a particle of common sense either, if you follow directions.) We can bring you 13 respectable and intensely fashionable people (if you will purchase excursion tickets for the party), who will declare to you the efficacy of the cure. But you want cures—want to raise merry Ned with those warts, 'tis supposed ; in that case, if this does not fix them, capture a strange black cat, at midnight (you'll find it jolly exercise), hold him up by the tail—and if he don't kick or scratch every confounded wart off your hand before you can apply the remedy, proceed as follows : Bite the extreme end of his tail off, (meanwhile look out for your eyes—the process is said to be hazardous, but somewhat amusing—and dot every wart on you hand with a drop of poor kitty's blood ; then let him go. Not a word must be spoken by either party, else will the charm be broken. Cannot tell how soon the warts will disappear, but am assured they will. Should both cures fail, write us, and we will make further inquiries.

